# Health Benefits of the Alkaloids from Lobeira (*Solanum lycocarpum* St. Hill): A Comprehensive Review

**DOI:** 10.3390/plants13101396

**Published:** 2024-05-17

**Authors:** Felipe Tecchio Borsoi, Glaucia Maria Pastore, Henrique Silvano Arruda

**Affiliations:** Departamento de Ciência de Alimentos e Nutrição, Faculdade de Engenharia de Alimentos (FEA), Universidade Estadual de Campinas (UNICAMP), Rua Monteiro Lobato nº 80, Campinas 13083-862, São Paulo, Brazil; felipe.tecchio@gmail.com (F.T.B.); glaupast@unicamp.br (G.M.P.)

**Keywords:** fruta-do-lobo, steroidal glycoalkaloids, solasonine, solamargine, biological activities, Solanaceae

## Abstract

*Solanum* is the largest genus within the Solanaceae family and has garnered considerable attention in chemical and biological investigations over the past 30 years. In this context, lobeira or “fruta-do-lobo” (*Solanum lycocarpum* St. Hill), a species predominantly found in the Brazilian Cerrado, stands out. Beyond the interesting nutritional composition of the fruits, various parts of the lobeira plant have been used in folk medicine as hypoglycemic, sedative, diuretic, antiepileptic, and antispasmodic agents. These health-beneficial effects have been correlated with various bioactive compounds found in the plant, particularly alkaloids. In this review, we summarize the alkaloid composition of the lobeira plant and its biological activities that have been reported in the scientific literature in the last decades. The compiled data showed that lobeira plants and fruits contain a wide range of alkaloids, with steroidal glycoalkaloid solamargine and solasonine being the major ones. These alkaloids, but not limited to them, contribute to different biological activities verified in alkaloid-rich extracts/fractions from the lobeira, including antioxidant, anti-inflammatory, anticancer, antigenotoxic, antidiabetic, antinociceptive, and antiparasitic effects. Despite the encouraging results, additional research, especially toxicological, pre-clinical, and clinical trials, is essential to validate these human health benefits and ensure consumers’ safety and well-being.

## 1. Introduction

The Solanaceae family is a large and varied family of trees, shrubs, and herbs including 90 genera and more than 2000 species. *Solanum* is the largest genus within the Solanaceae family and has garnered considerable attention in chemical and biological investigations over the past 30 years [1]. In this context, the lobeira or “fruta-do-lobo” (*Solanum lycocarpum* St. Hill), a species native to South America and predominantly found in the Brazilian Cerrado, stands out. Lobeira is known for being resistant and adaptable to dry and hot environments, making it a good food source for animals, especially the Lobo-guará (*Chrysocyon brachyurus*), particularly during the dry season when the availability of other foods is low [2]. This fruit has an interesting nutritional composition, as it has low levels of carbohydrates (10.97%), lipids (0.86%), and energy (57.1 kcal/100 g), demonstrating the potential for inclusion in restrictive diets (e.g., low-energy diets for obese individuals). Minerals such as potassium, magnesium, and calcium are predominant in its fruits (400.0, 8.4, and 14.7 mg/100 g, respectively), while other minerals like copper, manganese, iron, sodium, and zinc are present in low quantities (0.3, 0.095, 0.3, 0.66, and 0.179 mg/100 g, respectively). Also, lobeira is an excellent source of vitamin C providing more than 100% of Recommended Daily Intake (RDI) in only one serving (100 g of fresh edible fruit part) [2,3]. Beyond the nutritional aspects of the fruit, different parts of the lobeira plant have been used in folk medicine as a hypoglycemic, sedative, diuretic, antiepileptic, and antispasmodic agent [4,5,6]. Recent studies have shown that these health-beneficial effects may be related to different classes of bioactive compounds present in the lobeira plant, particularly alkaloids [2].

Alkaloids are secondary metabolites found in plants and some animals, containing nitrogen as a characteristic element present in their chemical structures [4]. In plants, these phytochemicals can accumulate in different organs (e.g., roots, fruits, leaves, tubers, etc.), playing a crucial role in the plant’s defense against parasites, particularly fungi and bacteria [7]. Studies conducted with the lobeira plant have shown that these compounds mainly accumulate in its unripe fruits [8]. Alkaloid-rich extracts obtained from lobeira fruit and their isolated alkaloids have attracted growing attention from researchers due to their wide range of pharmacological properties, including hypoglycemic, anticarcinogenic, and antiparasitic effects [2].

Despite several studies showing the potential health benefits of lobeira alkaloids, there is no literature review compiling data related to identifying and quantifying alkaloids and their associated biological activities. Therefore, this comprehensive review was designed to summarize the scientific data found in the literature in the last decades on the alkaloid composition of the lobeira plant and its biological activities to compile up-to-date information that will help clarify the advances made so far and the gaps to be filled by future studies. Thus, this review can be a reference material to support other researchers in conducting future studies on the isolation processes and applications of lobeira alkaloids.

## 2. Search Strategy and Studies Selection

In the current comprehensive review study, electronic searches were carried out using the main repositories of the world’s scientific data (Scopus, Google Scholar, Science Direct, Web of Science, and PubMed databases) to identify relevant studies published in scientific journals from 2001 to the present. We used the following terms to perform our bibliographic research: “Lobeira” OR “*Solanum lycocarpum*” AND “Alkaloids”. The abovementioned terms were searched on the article title, abstract, and keywords. The search was not restricted to any specific language. The studies that met the search criteria were selected for full-text review. Theses, editorials, communications, and conference abstracts were excluded. The inclusion criteria were studies that reported results concerning: (1) alkaloid-rich extracts or fractions from fruit or plant, (2) alkaloid composition, and (3) biological properties.

## 3. Lobeira (*Solanum lycocarpum* St. Hill)

### 3.1. Taxonomy

Scientific classification of lobeira taken from the SiBBr (Sistema de Informação Sobre a Biodiversidade Brasileira) [9].

**Kingdom:** Plantae

**Phylum:** Tracheophyta

**Class:** Magnoliopsida

**Order:** Solanales

**Family:** Solanaceae

**Genus:** *Solanum*

**Species:** *Solanum lycocarpum* St. Hill

### 3.2. Botanical Information

**Figure 1:**
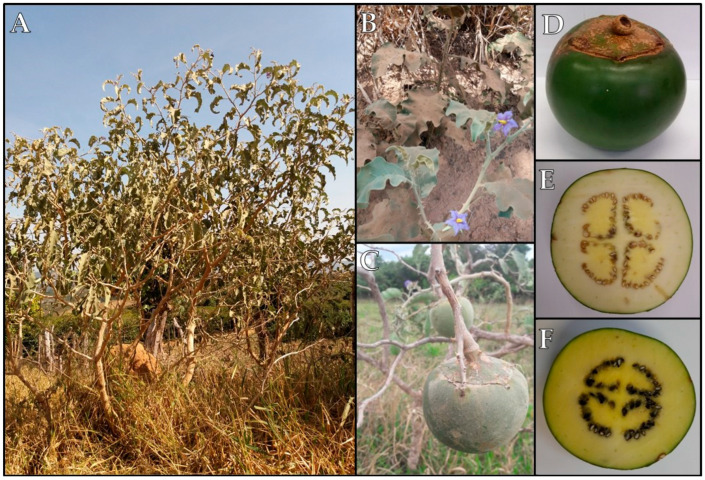
Lobeira (*Solanum lycocarpum* St. Hill): (**A**) tree, (**B**) leaves and flowers, (**C**) fruit in the plant, (**D**) fruit, (**E**) unripe fruit cross-section, and (**F**) ripe fruit cross-section. Photos taken by Gabrielle Silvano Arruda (Picture (**C**)) and Ana Paula Aparecida Pereira (Pictures (**A**,**B**,**D**–**F**)).

*Solanum lycocarpum* St. Hill is commonly known as lobeira, fruta-de-lobo, jurubebão, juripeba, and baba-de-boi, naturally occurring throughout tropical and subtropical Brazil, with a predominant presence in Cerradões, Cerrado, and Campo Cerrado environments. This species is widely distributed along the Brazilian territory, covering the states of Minas Gerais, São Paulo, Goiás, Bahia, Mato Grosso, Mato Grosso do Sul, Tocantins, Maranhão, Piauí, Rio de Janeiro, Paraná, and Federal District [3,10]. The lobeira plant is a shrub and tree species with evergreen or perennial leaf-changing behavior, capable of reaching up to 5 m in height (Figure 1A). It features a twisted cylindrical trunk with a rounded and open canopy measuring 3 to 4 m in diameter. Its branches are twisted, equipped with strong prickles, and covered with whitish or slightly ferruginous trichomes [10].

Lobeira leaves are porous, have a leathery texture, a greenish-gray color, and are covered by a tomentose layer (Figure 1B) [10,11]. The dimensions of the leaves vary, with lengths ranging from 6 to 24 cm and widths from 4 to 14 cm. The venation is broquidodromous, with prominent veins visible on both sides of the leaves and having a yellowish tone in contrast to the blade [10]. The bases of the leaves can have asymmetric, cordate, rounded, or obtuse shapes, while the apices are acute, rounded, or retuse. The petioles have a maximum length of up to 7 cm and are adorned with small recurved yellowish bristles, without the presence of stipules. They exhibit white pilosity on both sides, which becomes shiny in the sun [10,12].

The inflorescences are terminal or extrafoliaceous cymose, unbranched, covered with trichomes similar to those of the branches, with up to eight flowers. The flowers are hermaphroditic with a calyx of up to 1.7 cm in length, having deeply divided lanceolate lobes, covered abaxially with cinereous-white trichomes and prickly acaulescent structures (Figure 1B). The corolla is rotated, displaying blue, lilac, or bluish-violet coloration, with lobes divided up to the middle portion, measuring up to 2.1 cm in length, featuring large yellow stamens [10,13].

The fruits are indehiscent, fleshy, berry-type, globular, and polyspermous, measuring 7–16 cm in diameter and weighing between 400 and 900 g [10]. The peel is tomentose, with small hairs that easily detach upon touch and remain green even after ripening (Figure 1C,D). On the other hand, the pulp has a firm texture and appears white when unripe (Figure 1E), turning yellow with a soft texture, sweet taste, and an extremely aromatic aroma when fully ripe (Figure 1F) [14]. Each fruit can contain 300 to 500 seeds (Figure 1E,F) [10]. The seeds are ellipsoid or sub-ellipsoid in shape, albuminous, and have a circinate embryo. Most seeds have an average length of 6–7 mm, a width of 4.58 to 5.08 mm, and a thickness of 1.50 to 2.10 mm [10,14].

The flowering and fruiting cycle of lobeira can vary depending on the geographical region, climate, and local conditions. For example, in the state of Paraná, flowering can occur from March to December. In the state of Piauí, it occurs from April to September, and in the state of São Paulo, from September to December. Furthermore, in the state of Minas Gerais, this species has shown continuous flowering throughout the entire year, with brief intervals between reproductive phases. Fruit development, on the other hand, takes place after the successful pollination of the flowers. For example, in Piauí, fruiting occurs from June to July, in São Paulo from May to July, and in Paraná from November to June [10].

Lobeira fruit has low pH (4.87) and total titratable acidity (0.79 g citric acid/100 g), and high values of total soluble solids (24.0 °Brix) and soluble solids to titratable acidity ratio (30.38). These characteristics are highly desirable for its application in some processed food products, especially jams, yogurts, and sweets [3,15,16].

## 4. Alkaloids Found in Lobeira

The results of the chemical characterization studies conducted on lobeira alkaloids are summarized in Table 1. All studies focused only on fruit characterization. Steroidal alkaloids are the major class of alkaloids identified in this species. Furthermore, calystegine alkaloids have also been reported in some studies.

**Figure 2:**
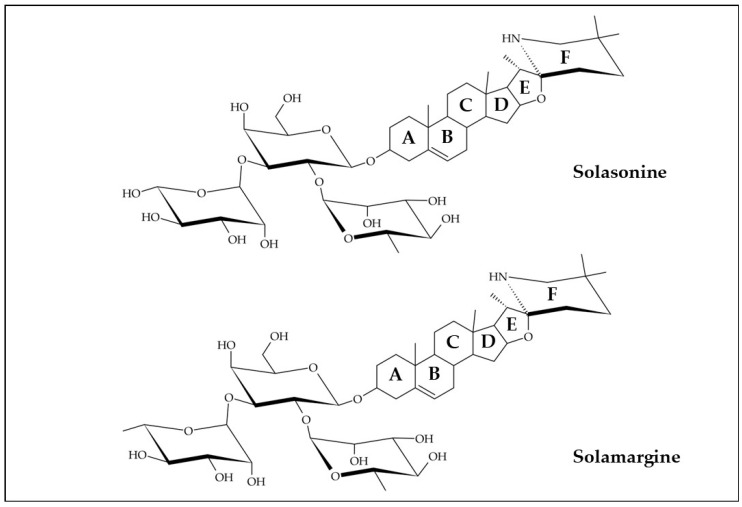
Chemical structure of the main steroidal glycoalkaloids found in the lobeira fruit: solasonine and solamargine. The letters A–F refer to the order of the rings present in the structures of steroidal glycoalkaloids. Own authorship created by ChemSketch software version 2021.2.1.

The steroidal glycoalkaloids solamargine and solasonine (Figure 2) are the major alkaloid compounds in the lobeira, as illustrated in Table 1. Steroidal glycoalkaloids are secondary metabolites found in some plants, especially in the Solanaceae family. These alkaloids are characterized by the presence of a steroidal nucleus with four interconnected rings, similar to the structure of steroids. Furthermore, sugar groups can attach to different positions in the steroidal backbone, typically in the A ring, resulting in glycosides formation [33,34]. The amount of steroidal glycoalkaloids such as solamargine and solasonine is influenced by the ripening stage (unripe or ripe fruit) as well as the method of obtaining these extracts. Tiossi et al. [8] did not detect any amount of these alkaloids in the branches and leaves of the lobeira plant. However, it was observed that unripe fruits exhibited significantly higher concentrations of glycoalkaloids compared to the ripe fruit, providing, respectively, 1.04% and 0.83% of solasonine and 0.69% and 0.60% of solamargine. This can be explained by the fact that these compounds play a crucial role in the defense response of plants in the Solanaceae family against a wide range of pathogens and predators during the ripening process of their fruits [33]. The exact reason for the reduction in these glycoalkaloids during fruit ripening is not known. However, it has been reported that high levels of glycoalkaloids make the fruit more toxic and less palatable. Therefore, the reduction in these compounds during fruit ripening is essential for the fruits to become edible for frugivorous animals, allowing the spread of mature seeds and, consequently, the propagation of the species [8].

Tiossi et al. [8] verified that different extraction protocols result in different selectivity for obtaining steroidal glycoalkaloids from lobeira fruits. While the dried fruit biomass and its hydroalcoholic extract (80% ethanol) contain around 2% and 11% of glycoalkaloids, respectively, the alkaloid-selective extract recovered approximately 90% (45.09% solasonine and 44.37% solamargine). Similarly, Martins et al. [23] found that the hydroethanolic extract (96% ethanol) contained less than 5% of solasonine (4.6%) and solamargine (4.4%), while a semi-purification process (hydroethanolic fraction containing 40% ethanol) increased these percentages to 15.3% and 35.7%, respectively. Furthermore, after the purification and isolation of these compounds, the yields reached 71.6% for solasonine and 63.1% for solamargine. It has been demonstrated by other studies that the recovery of solasonine and solamargine is less than 7% with hydroethanolic solutions alone (80% ethanol) [21,22]. Therefore, the purification of crude extracts is necessary to obtain alkaloid-rich extracts; for example, the acid–base selective extraction process can achieve alkaloid yields of approximately 90% (42% solasonine and 47% solamargine) [26,27,28,29,30].

As can be seen in Table 1, an acid–base selective extraction protocol was used to obtain extracts rich in steroidal glycoalkaloids from the lobeira fruit. Through this purification protocol, it was possible to obtain extracts with approximately 90% steroidal glycoalkaloids solamargine and solasonine in practically equivalent quantities. Although steroidal glycoalkaloids solamargine and solasonine are the main alkaloids present in lobeira fruit, other steroidal alkaloids, glycosylated or not, have also been found and identified (Table 1). Pereira et al. [24] investigated and identified in an alkaloid extract five steroidal glycoalkaloids (dihydroxysolamargine, three isomers of hydroxysolamargine, and solasonine). Subsequently, in another more in-depth study, Pereira et al. [17] identified 27 steroidal glycoalkaloids in different parts of the fruit, such as peel, pulp, and seed. Furthermore, their findings allowed to observe that the unripe fruit fractions were richer in alkaloid compounds. However, during the ripening process, these compounds had a significant reduction of up to 85%, as was the case with solamargine in the pulp. Morais et al. [18] found 21 steroidal alkaloid derivatives in ethanolic extract of ripe fruit, among them 19 steroidal glycoalkaloids (robeneoside B or hydroxysolasonine isomers, solanandaine isomers, unknown steroidal glycosylated alkaloids, and khasianine or β_2_-solanine isomer) and 2 non-glycosylated steroidal alkaloids (peiminine and solasodine). Subsequently, Morais et al. [19] fractionated this ethanolic extract into ethyl acetate and hydroethanolic fractions and identified 13 alkaloid compounds in these fractions, including 11 steroidal glycoalkaloid derivatives (robeneoside B or hydroxysolasonine isomers, solanandaine isomers, unknown steroidal glycosylated alkaloids, and khasianine or β_2_-solanine isomer) and 2 non-glycosylated steroidal alkaloids (peiminine and solasodine). In another study, Morais et al. [20] evaluated the profile of alkaloids present in the dichloromethane fraction obtained from the ethanolic extract and found 13 steroidal alkaloids, among which 10 steroidal glycoalkaloids (solasonine, solamargine, and unknown steroidal glycoalkaloids) and 3 non-glycosylated steroidal alkaloids (peiminine or imperialine, peimine or imperialine, and solasodine). Yoshikawa et al. [25] isolated five steroidal glycoalkaloids (robeneosides A and B, solamargine, solasonine, and 12-hydroxysolasonine) from the lobeira fruit. Subsequently, Nakamura et al. [6] identified 11 steroidal glycoalkaloids, namely, lyconosides Ia, Ib, II, III, and IV, robeneosides A and B, solamargine, solasonine, 12-hydroxysolasonine, and lobofrutoside). These compounds were isolated from methanolic extracts of lobeira fruits, and their structures were determined based on chemical and physicochemical evidence, providing further insight into the biosynthesis of these compounds.

Beyond alkaloids belonging to the classes of steroidal alkaloids found in the lobeira fruit reported above, calystegines (calystegines A_3_, B_1_, B_2_, and C_1_) were observed in the fruits for the first time by Souto et al. [32]. Among these, calystegine B_2_ was the major alkaloid, accounting for 48.34 mg/kg of fresh weight [32]. Calystegines are a group of polyhydroxy alkaloids with a nortropane skeleton functionalized by three to five hydroxyl groups. This class of alkaloids is found in various plants, including those of the Solanaceae family [35]. Calystegines are known for their potential toxicity to humans, especially when consumed in large quantities. On the other hand, calystegines may have inhibitory effects on certain glycosidase enzymes [32,36]. Therefore, their precise physiological and biochemical roles in plants and their potential health effects on humans are still areas of active research.

These data show that the lobeira fruit is rich in alkaloids, especially steroidal glycoalkaloids, with solamargine and solasonine being its major components. In addition, there is a reduction in these compounds during the fruit ripening, mainly in the pulp. Thus, unripe fruits can be potential sources for isolating and purifying steroidal glycoalkaloids with biological properties for application in foods, pharmaceuticals, and cosmetics.

## 5. Biological Activities Reported for Alkaloids from Lobeira

Lobeira fruit has rarely been employed as a food source for humans, mainly used in the preparation of jams and sweets. However, both the fruit and plant parts have been utilized for centuries in folk medicine as hypoglycemic, hypocholesterolemic, antiepileptic, diuretic, sedative, and antispasmodic [2]. The presence of various alkaloids in the fruit and plant parts of lobeira, particularly solasonine and solamargine (as summarized above), may contribute to a wide range of effects on human health. Therefore, numerous studies have been conducted in recent years to validate the reported effects of folk medicine and explore other biological activities and therapeutic effects of the lobeira, particularly alkaloid-rich extracts and fractions or even some alkaloids purified from these fractions (see Table 2). In the sections below, the main biological activities reported so far for the alkaloids found in the lobeira, emphasizing the identification of their biological targets and the discovery of their molecular mechanisms based on in vitro and in vivo studies are presented and discussed.

### 5.1. Antioxidant Activity

Some in vitro studies have demonstrated that alkaloid-rich extracts and/or fractions obtained from the lobeira fruit exhibit antioxidant activity in different assays (see Table 2). Morais et al. [18] reported a high antioxidant activity in an alkaloid-rich extract obtained from ripe fruit. The evaluated extract (ethanolic extract) showed antioxidant activity in the DPPH assay slightly higher than the positive control BHT, with IC_50_ values of 14.37 and 16.36 µg/mL, respectively. The authors found that this extract was particularly composed of steroidal alkaloids (21 compounds) and phenolic compounds derived from caffeic and coumaric acids (8 compounds). In another study, Morais et al. [19] evaluated the antioxidant activity of hexane, ethyl acetate, and hydroethanolic fractions obtained from the ethanolic extract of ripe fruit using DPPH and FRAP assays. Among the analyzed fractions, the ethyl acetate fraction exhibited the highest antioxidant activities in both methods employed. The ethyl acetate fraction had antioxidant activity in the DPPH assay (IC_50_ value of 1.02 µg/mL) much higher than in the BHT (IC_50_ value of 16.36 µg/mL) and similar to ascorbic acid (IC_50_ value of 1.62 µg/mL), while the hexane and hydroethanolic fractions showed IC_50_ values close to BHT (19.91 and 18.87 µg/mL, respectively). Regarding the FRAP assay, the ethyl acetate fraction (IC_50_ value of 1.48 µg/mL) showed activity very similar to BHT (IC_50_ value of 1.19 µg/mL) and inferior to ascorbic acid (IC_50_ value of 0.76 µg/mL), whereas the other fractions showed antioxidant activity much lower than the positive controls BHT and ascorbic acid, with IC_50_ values of 22.15 and 19.12 µg/mL for the hexane and hydroethanolic fractions, respectively. Instrumental analyses demonstrated that the ethyl acetate fraction was rich in steroidal alkaloids (11 compounds) and phenolic compounds derived from caffeic and coumaric acids (16 compounds). Subsequently, Morais et al. [20] investigated the antioxidant activity of the dichloromethane fraction obtained from the ethanolic extract of ripe fruit using DPPH and FRAP assays. The dichloromethane fraction displayed antioxidant activity (IC_50_ value of 1.99 µg/mL) much higher than the BHT (IC_50_ value of 16.36 µg/mL) and similar to ascorbic acid (IC_50_ value of 1.62 µg/mL) by the DPPH assay but presented slightly lower antioxidant activity (IC_50_ value of 3.55 µg/mL) than positive controls (IC_50_ values of 0.76 and 1.19 µg/mL for ascorbic acid and BHT, respectively) by the FRAP assay. Analysis of the chemical composition demonstrated that this fraction was particularly composed of steroidal alkaloids (13 compounds) and phenolic compounds derived from caffeic and coumaric acids (4 compounds). Pereira et al. [17] studied the effect of the fruit ripening stage on the antioxidant activity of different fruit fractions (peel, pulp, and seed) using the ORAC assay and found that regardless of the analyzed fraction, antioxidant activity values were higher for the unripe fruit (964.07–1256.75 μmol TE/mg) than the ripe one (581.56–1145.89 μmol TE/mg). Furthermore, the seeds showed the highest antioxidant activity values at both ripening stages (1256.75 and 1145.89 μmol TE/mg for unripe and ripe fruits, respectively). Instrumental analyses of hydroethanolic extracts (70% ethanol) from these fruit fractions revealed the presence of 39 phenolic compounds (particularly compounds derived from caffeic and *p*-coumaric acids) and 27 steroidal alkaloids. However, the authors observed a reduction in alkaloid content throughout ripening that could be related to the decrease in antioxidant activity in ripe fruit. In fact, when performing a Pearson correlation analysis for antioxidant activity using the ORAC assay, the authors observed higher positive correlation values for alkaloids than for phenolic compounds. Macáková et al. [37] reviewed the antioxidant activity of more than 130 alkaloids isolated from plants, fungi, algae, bacteria, and animals or prepared from them. Some of these alkaloids showed the ability to scavenge the DPPH radical either similar to or even higher than antioxidant standards, demonstrating that this class of phytochemicals can be potent antioxidants.

### 5.2. Anticancer and Antigenotoxic Activities

Recent studies have demonstrated the anticancer potential of the alkaloid extract from the lobeira fruit (extract containing approximately 90% of the alkaloids solasonine and solamargine in nearly equivalent proportions) and the major alkaloids isolated from this extract (solasonine and solamargine) against different types of tumors (see Table 2). Munari et al. [38] investigated the antiproliferative activity of the alkaloid extract, solasonine, and solamargine against eight cancer cell lines, including one murine and seven human cell lines. Solamargine was the most active compound against all human cancer cell lines tested with IC_50_ values varying from 4.58 to 18.23 μg/mL, followed by solasonine (IC_50_ values varying from 6.01 to 26.21 μg/mL) and alkaloid extract (IC_50_ values varying from 9.60 to 40.82 μg/mL). Alkaloid extract and the isolated alkaloids exhibited antiproliferative activity similar to or greater than the well-known chemotherapeutic drugs camptothecin and etoposide for the majority of cancer cell lines, whose IC_50_ values were 5.71–36.09 and 2.18–325.40 μg/mL, respectively. HepG2 (human hepatocellular liver carcinoma) and HeLa (human cervical adenocarcinoma) cancer cell lines showed the highest susceptibilities to the lobeira fruit alkaloids, with IC_50_ values of 4.58–9.60 and 7.48–16.04 μg/mL, respectively. Furthermore, the alkaloid extract and the isolated alkaloids showed low toxicity against the normal cell lines V79 (Chinese hamster lung fibroblasts) and GM07492A (human lung fibroblasts), with IC_50_ values of 16.75–37.60 and 25.39–38.01 μg/mL, respectively. Munari et al. [38] found that the alkaloids solamargine and solasonine isolated from lobeira fruit exhibited a strong and similar cytotoxic effect against MCF-7 cells (human breast adenocarcinoma cells), with IC_50_ values of 18.23 and 22.25 μg/mL, respectively. Similar results were reported by Barbosa et al. [39] in the same cancer cell line (MCF-7 cells), where the solamargine and solasonine showed IC_50_ values of 13.55 and 14.57 μmol/L, respectively. Additionally, solamargine exhibited the lowest cytotoxicity against the normal cell lines B16 (murine skin, IC_50_ value of 34.075 μmol/L) and 3T3 (normal mouse embryo fibroblasts, IC_50_ value of 20.11 μmol/L).

Several studies evaluated the cytotoxic effect of the alkaloid extract from lobeira fruit, both in free form and encapsulated, in 2D and 3D models of bladder cancer cell line (RT4) [29,30,40,41]. The free alkaloid extract showed high cytotoxicity in both 2D and 3D models of RT4 cancer cells, with IC_50_ values ranging between 8.17 and 15.24 μg/mL for the 2D model [29,30,40,41] and 21.81 μg/mL for the 3D model [29]. Alkaloid extract exerted cytotoxic effects on RT4 cancer cells by inducing cellular apoptosis and causing cell cycle arrest at the S phase [40]. The encapsulation of the alkaloid extract and/or the functionalization of nanoparticles containing the alkaloid extract potentiated the cytotoxic effect against RT4 cancer cells in both 2D and 3D models by approximately two times compared to the free extract due to the higher internalization of the alkaloid extract-loaded nanoparticles by cancer cells [30,40,41]. Miranda et al. [41] observed that folic acid-functionalized polymeric nanoparticles loading alkaloid extract were about 2-fold as potent as the free extract against RT4 cancer cells, in addition to exhibiting an uptake by cancer cells about 2-fold higher than that noticed in normal cells (HaCaT cells). These effects can be explained by the increased selectivity of folic acid-functionalized polymeric nanoparticles loading alkaloid extract for cancer cells, as the presence of folic acid on the surface of these particles may enhance their absorption by cancer cells due to the overexpression of folate receptors on the membrane these cells [41]. In another study, Miranda et al. [29] investigated the cytotoxic and chemosensitizing effects of this alkaloid extract on bladder cancer cells (RT4) and patient-derived xenograft (PDX) bladder cancer cells. The researchers noted that the extract was able to reduce the viability of bladder cancer cells in both 2D and 3D models. Additionally, the alkaloid extract exhibited chemosensitizing effects by increasing the sensitivity of 2D and 3D cultures of RT4 and PDX cells to cisplatin. The combination of the alkaloid extract (5 μg/mL) with cisplatin (8.4 μmol/L) inhibited the formation and migration of RT4 cancer cell colonies and induced their apoptosis more efficiently than the agents separately. The reduction in RT4 cancer cell migration occurred through the downregulation of MMP-2 and MMP-9 expression, while apoptosis was induced by the downregulation of protein expression of Bcl-2, Bcl-xL, PARP, survivin, Cap-3, and Cap-9, and the upregulation of Bax.

When assessing the antiproliferative effect of the alkaloid extract from lobeira fruit and its isolated alkaloids (solamargine and solasonine) on the B16F10 cancer cell line (murine melanoma), Munari et al. [38] found that solamargine (IC_50_ value of 10.15 μg/mL) was the most potent anticancer agent, presenting a much lower IC_50_ value than that of known chemotherapeutic drugs, camptothecin (IC_50_ value of 20.17 μg/mL) and etoposide (IC_50_ value of 48.91 μg/mL). In a subsequent study, Furtado et al. [31] investigated the antimelanoma effect of free solamargine isolated from lobeira fruit and nanoparticles of yttrium vanadate functionalized with 3-chloropropyltrimethoxysilane containing solamargine using a syngeneic mouse melanoma model with B16F10 cell line. Mice treated subcutaneously with free solamargine (5 or 10 mg/kg bw) for 5 days showed a significant reduction in tumor size and the number of mitoses in the tumor tissue compared to the implanted control group, while nanoencapsulated solamargine (10 mg solamargine/kg bw) was able to reduce only the number of mitoses in the tumor tissue. Furthermore, treatments with both free and nanoencapsulated solamargine significantly reduced the frequency of hepatic DNA damage compared to the implanted control group, without causing apparent signs of systemic toxicity, nephrotoxicity, and genotoxicity, suggesting that solamargine may be considered a promising candidate in cancer therapy with no apparent toxic effects.

Munari et al. [27] showed the promising anticancer properties of the alkaloid extract from lobeira fruit in a DMH-induced colon cancer animal model. The authors found that oral administration of the alkaloid extract (15, 30, and 60 mg/kg bw) for 4 weeks significantly reduced the DMH-induced number of aberrant foci crypt and aberrant crypts in the distal colon of the rats.

Cancer originates as a result of mutations in normal cells leading to specific phenotypes, including immortality, hyperproliferation, and invasion into normal tissues, among others [42]. Therefore, DNA mutations play a key role in the onset and progression of cancer. Several studies conducted with cellular and animal models have demonstrated the antigenotoxic potential of alkaloid-rich extracts/fractions obtained from lobeira fruit and its isolated alkaloids, without causing cytotoxic and/or genotoxic effects (see Table 2).

Hydroethanolic extract from the unripe lobeira fruit (80% ethanol; 6.57% solasonine and 4.60% solamargine) was tested for its cytotoxic, genotoxic, and antigenotoxic properties in Chinese hamster lung fibroblast cells (V79 cells) treated with methyl methanesulfonate (MMS) and doxorubicin (DXR). The results demonstrated the absence of cytotoxic and genotoxic effects for extract concentrations up to 64 µg/mL [21,22]. On the other hand, this extract (16–64 µg/mL) exhibited antigenotoxic effects by reducing both the MMS-induced frequency of micronuclei and DNA damage [21], as well as the DXR-induced frequencies of chromosomal aberrations, abnormal metaphases, and the number of cells with aberrations [22]. Subsequent studies also evaluated the cytotoxic, genotoxic, and antigenotoxic effects of the alkaloid extract from lobeira fruit, as well as its isolated alkaloids, in MMS-treated Chinese hamster lung fibroblast cells (V79 cells) [26,43]. Alkaloid extract displayed cytotoxic and genotoxic effects only at concentrations above 32 µg/mL [26]. On the other hand, the alkaloids solamargine (1.78–7.1 μg/mL) and solasonine (3.6–14.4 μg/mL) did not show any genotoxic effects at the tested concentrations, but they were cytotoxic at concentrations higher than 14.2 and 28.8 μg/mL, respectively [43]. Meanwhile, both the alkaloid extract (8–32 µg/mL) and the alkaloids solamargine (1.78–7.1 μg/mL) and solasonine (3.6–14.4 μg/mL) had an antigenotoxic effect, protecting V79 cells against MMS-induced genomic and chromosomal damages [26,43]. Studies conducted in animal models have confirmed the absence of cytotoxic and genotoxic effects as well as the antigenotoxic potential of alkaloid-rich extracts from the lobeira fruit [22,27,44]. Vieira et al. [44] verified that the hydroethanolic extract from unripe fruits (96% ethanol) did not exert genotoxic effects at any of the tested doses (5, 10, 25, 50, or 80 mg/kg bw administrated intraperitoneally), maintaining the frequency of micronucleated polychromatic erythrocytes in bone marrow cells of mice. Furthermore, all tested doses of this extract (5–80 mg/kg bw) were able to mitigate the genotoxic action of mitomycin C (MMC) by reducing the MMC-induced frequency of micronucleated polychromatic erythrocytes in bone marrow cells of mice. Similar results were reported by Tavares et al. [22] for another hydroethanolic extract from unripe fruits (80% ethanol; 6.57% of solasonine and 4.60% of solamargine). The researchers observed that the oral administration of this extract (0.25, 0.50, 1.0, and 2.0 g/kg bw) did not promote any cytotoxic and genotoxic effects in mice, while simultaneously protecting the animals from the genotoxic action of doxorubicin. Likewise, Munari et al. [27] noted that the oral administration of the fruit alkaloid extract (45% solasonine and 44% solamargine) for 14 days was incapable of promoting cytotoxic and genotoxic effects in mice at any tested doses (15, 30, and 60 mg/kg bw). Furthermore, this extract displayed antigenotoxic properties by reducing the MMS-induced frequency of micronucleated polychromatic erythrocytes in bone marrow cells and DNA damage in the liver cells of mice.

In summary, it has been demonstrated that the lobeira fruit alkaloids may exert their antigenotoxic activity by reducing genomic and chromosomal damage induced by toxic agents in normal cells, inhibiting the migration of cancer cells, and inducing apoptosis in cancer cells.

**Table 2:** A summary of studies showing the biological activities of alkaloids and alkaloid-rich extracts from lobeira.

Bioactivity	Plant Part	Extract Type	Method/Model	Major Findings	Ref.
Antioxidant	Unripe and ripe fruits	Hydroethanolic extract (70% ethanol)	ORAC-based in vitro assay	All fractions (pulp, peel, and seeds) of the unripe fruit (964.07–1256.75 μmol TE/mg) exhibited higher antioxidant activity than in the ripe fruit (581.56–1145.89 μmol TE/mg). Seeds showed the highest antioxidant activity (1256.75 and 1145.89 μmol TE/mg), followed by pulp (1086.61 and 581.56 μmol TE/mg), and peel (964.07 and 914.27 μmol TE/mg).	[17]
	Ripe fruit	Ethanolic extract	DPPH-based in vitro assay	Extract showed high antioxidant activity with IC_50_ value (14.37 µg/mL) very close to the positive control BHT (16.36 µg/mL).	[18]
	Ripe fruit	Ethyl acetate and hydroethanolic fractions from ethanolic extract	DPPH- and FRAP-based in vitro assays	Ethyl acetate fraction showed the highest antioxidant activity with IC_50_ values (1.02 and 1.48 µg/mL for DPPH and FRAP assays, respectively) very close to the positive control ascorbic acid (1.62 and 0.76 µg/mL for DPPH and FRAP assays, respectively). Hydroethanolic fraction showed IC_50_ values of 18.87 and 19.12 µg/mL for DPPH and FRAP assays, respectively.	[19]
	Ripe fruit	Dichloromethane fraction from ethanolic extract	DPPH- and FRAP-based in vitro assays	Dichloromethane fraction showed high antioxidant activity with IC_50_ values (1.99 and 3.55 µg/mL for DPPH and FRAP assays, respectively) very close to the positive control ascorbic acid (1.62 and 0.76 µg/mL for DPPH and FRAP assays, respectively).	[20]
Antigenotoxic	Unripe fruit	Hydroethanolic extract (80% ethanol; 6.57% of solasonine and 4.60% of solamargine)	MMS-treated Chinese hamster lung fibroblast cells (V79)	No cytotoxic and genotoxic effects at concentrations ≤64 µg/mL. ↓ MMS-induced frequency of micronuclei and DNA damage (16–64 µg/mL).	[21]
	Unripe fruit	Hydroethanolic extract (80% ethanol; 6.57% of solasonine and 4.60% of solamargine)	Doxorubicin-treated Chinese hamster lung fibroblast cells (V79)	No cytotoxic and genotoxic effects at concentrations ≤64 µg/mL, maintaining the mitotic index and not causing chromosomal aberrations. ↓ Doxorubicin-induced frequencies of chromosomal aberrations, abnormal metaphases, and number of cells with aberrations (16–64 µg/mL).	[22]
	Unripe fruit	Hydroethanolic extract (80% ethanol; 6.57% of solasonine and 4.60% of solamargine)	Doxorubicin-induced Swiss mice treated intragastrically with hydroethanolic extract (0.25, 0.50, 1.0, and 2.0 g/kg bw)	Extract did not show genotoxic effects at any of the tested doses, maintaining the frequency of micronucleated polychromatic erythrocytes in bone marrow cells. Extract did not show cytotoxic effects at any of the tested doses, maintaining the number of polychromatic erythrocytes in relation to the total number of erythrocytes in bone marrow cells. All doses reduced the doxorubicin-induced frequency of micronucleated polychromatic erythrocytes in bone marrow cells.	[22]
	Unripe fruit	Hydroethanolic extract (96% ethanol)	MMC-induced Swiss mice treated intraperitoneally with hydroethanolic extract (5, 10, 25, 50, or 80 mg/kg bw)	Extract did not show genotoxic effects at any of the tested doses, maintaining the frequency of micronucleated polychromatic erythrocytes in bone marrow cells. Extract showed cytotoxic effects at all tested doses, reducing the number of polychromatic erythrocytes in relation to the total number of erythrocytes in bone marrow cells. All doses reduced the MMC-induced frequency of micronucleated polychromatic erythrocytes in bone marrow cells.	[44]
	Fruit	Alkaloid extract (45.09% of solasonine and 44.37% of solamargine)	MMS-treated Chinese hamster lung fibroblast cells (V79)	No cytotoxic and genotoxic effects at concentrations ≤32 µg/mL. ↓ MMS-induced DNA damage and frequency of cells with chromosomal aberrations (8–32 µg/mL).	[26]
	Fruit	Alkaloid extract (45% of solasonine and 44% of solamargine)	MMS-induced Swiss mice treated intragastrically with alkaloid extract (15, 30, and 60 mg/kg bw) for 14 days	Extract did not show genotoxic effects at any of the tested doses for the three different collection periods (24 h, 7 days, and, 14 days), maintaining the frequency of micronucleated polychromatic erythrocytes in bone marrow cells and not causing DNA damage in liver cells. Extract did not show cytotoxic effects at any of the tested doses, maintaining the number of polychromatic erythrocytes in relation to the total number of erythrocytes in bone marrow cells. All doses reduced the MMS-induced frequency of micronucleated polychromatic erythrocytes in bone marrow cells and DNA damage in liver cells.	[27]
	Fruit	Solasonine and solamargine isolated from lobeira	MMS-treated Chinese hamster lung fibroblast cells (V79)	Solamargine and solasonine showed cytotoxicity in V79 cells at concentrations higher than 14.2 and 28.8 μg/mL, respectively. Both alkaloids did not show genotoxic effects at the evaluated concentrations, maintaining the frequencies of micronuclei and not causing DNA damage and chromosomal aberrations. Solamargine (1.78–7.1 μg/mL) and solasonine (3.6–14.4 μg/mL) reduced the MMS-induced DNA damage and frequency of chromosomal aberrations. Both alkaloids were unable to modulate the genotoxicity induced by camptothecin and etoposide.	[43]
Anticancer	Fruit	Alkaloid extract (AE) (42.86% of solasonine and 47.96% of solamargine) and natural lipid-based nanoparticles loading AE (NLN-AE)	Bladder cancer cells (RT4)	AE reduced the cell viability of bladder cancer cells in a dose-dependent manner (IC_50_ 15.24 μg/mL). NLN-AE reduced the cell viability of bladder cancer cells in a dose-dependent and time-dependent manner. NLN-AE showed higher cytotoxicity than free AE after 72 h of treatment and induced apoptosis.	[30]
	Fruit	Alkaloid extract (AE) (42.86% of solasonine and 47.96% of solamargine) and nanoparticles loading AE (NP-AE)	Bladder cancer cells (RT4)	NP-AE was more potent than the free extract after 24 h of incubation in 2D model (IC_50_ 4.18 and 8.17 μg/mL, respectively). NP-AE displayed higher cytotoxicity than the free extract (about 2-fold higher) in 2D and 3D models. Bladder cancer cells cultured under 3D conditions exhibited a higher resistance to the treatments (IC_50_ about 3-fold higher than in 2D cell culture). Treatments induced apoptosis and cell cycle arrest in the S phase.	[40]
	Fruit	Alkaloid extract (AE) (42.86% of solasonine and 47.96% of solamargine) and folic acid-functionalized polymeric nanoparticles loading AE (FNP-AE)	Bladder cancer cells (RT4)	FNP-AE (IC_50_ 3.78 and 7.7 μg/mL for 2D and 3D models, respectively) was about 2-fold as potent as the free extract. Bladder cancer cells cultured under 3D conditions exhibited a higher resistance to the treatments (IC_50_ about 2-fold higher than in 2D cell culture). The uptake of FNP-AE was 2-fold higher in cancer cells than normal cells (HaCaT cells).	[41]
	Fruit	Alkaloid extract (42.86% of solasonine and 47.96% of solamargine)	Bladder cancer cells (RT4) and patient-derived xenografts (PDX) bladder cancer cells	↓ RT4 and PDX cells viability in 2D (IC_50_ 10.12 and 38.21 μg/mL) and 3D (IC_50_ 21.86 and 54.43 μg/mL) models. ↑ Sensitivity of 2D and 3D cultures of RT4 and PDX cells to cisplatin. Extract plus cisplatin inhibited colony formation (40%) and migration (28.38%) and induced apoptosis (57%) in RT4 cells. Extract plus cisplatin downregulated the expression of Bcl-2, Bcl-xL, PARP, survivin, Cap-3, Cap-9, MMP-2, and MMP-9, and upregulated the expression of Bax, in RT4 cells.	[29]
	Fruit	Alkaloid extract (45% of solasonine and 44% of solamargine)	DMH-induced colon cancer in Wistar rats treated intragastrically with alkaloid extract (15, 30, and 60 mg/kg bw) for 4 weeks	All doses reduced the DMH-induced number of aberrant foci crypt and aberrant crypts in the distal colon.	[27]
	Fruit	Alkaloid extract, solasonine, and solamargine	Murine melanoma (B16F10), human colon carcinoma (HT29), human breast adenocarcinoma (MCF-7), human cervical adenocarcinoma (HeLa), human hepatocellular liver carcinoma (HepG2), and human glioblastoma (MO59J, U343, and U251) cells	Solamargine was the most potent antitumor compound (IC_50_ 4.58–18.23 μg/mL), followed by solasonine (IC_50_ 6.01–26.21 μg/mL) and alkaloid extract (IC_50_ 9.60–40.82 μg/mL). IC_50_ values were lower or similar to known chemotherapy drugs (camptothecin (IC_50_ 5.71–36.09 μg/mL) and etoposide (IC_50_ 2.18–325.40 μg/mL)) for most tumor cells. HepG2 and HeLa were the tumor cells more sensitive to the treatments (IC_50_ 4.58–9.60 and 7.48–16.04 μg/mL, respectively).	[38]
	Fruit	Solasonine and solamargine isolated from lobeira fruit	Human breast adenocarcinoma cells (MCF-7)	Both alkaloids showed similar antitumor activity against MCF-7 cells (IC_50_ 13.55 and 14.57 μmol/L for solamargine and solasonine, respectively).	[39]
	Fruit	Solamargine and YVO_4_:Eu^3+^:CPTES:SM	Syngeneic C57BL/6 mouse melanoma model (B16F10 cells) treated subcutaneously with solamargine (5 or 10 mg/kg bw) and YVO_4_:Eu^3+^:CPTES:SM (10 mg solamargine/kg bw) for 5 days	Solamargine reduced tumor size and frequency of mitoses in tumor tissue. YVO_4_:Eu^3+^:CPTES:SM reduced the number of mitoses in tumor tissue. Solamargine (10 mg/kg bw) and YVO_4_:Eu^3+^:CPTES:SM reduced hepatic DNA damage. No apparent signs of systemic toxicity, nephrotoxicity, and genotoxicity initiated by treatments either with solamargine alone or YVO4:Eu3+:CPTES:SM.	[31]
Antiparasitic	Leaves	Infusion at room temperature	In vitro antileishmanial activity against promastigotes forms of *Leishmania guyanensis* (strain AMC2014), *L. major* (strain MHOM/IR/1972/NADIM5), and *L. donovani* (strain GEDII) and intracellular (THP-1 human acute monocytic leukemia cells) amastigotes form of *L. donovani* (strain BHU814)	Extract was more active against promastigotes forms (IC_50_ 16–61 μg/mL). *L. donovani* GEDII promastigotes was the most sensitive strain to the extract (IC_50_ 16 μg/mL). Extract was only moderately active against *L. donovani* BHU814 amastigotes (IC_50_ 374 μg/mL). Extract had low cytotoxicity on THP-1 cells (IC_50_ > 500 μg/mL).	[45]
	Fruit	Hydroethanolic extract (96% ethanol; 4.6% of solasonine and 4.4% of solamargine), solasonine, solamargine, and solasodine	In vitro antileishmanial activity against *Leishmania infantum* promastigotes and intracellular (mouse peritoneal macrophages) amastigotes forms	Solasodine was the most active compound against promastigote forms (IC_50_ 4.7 μg/mL), followed by solamargine, hydroethanolic extract, and solasonine (IC_50_ 8.1, 16.7, and 22.7 μg/mL, respectively). Solasonine and solamargine showed high anti-*L. infantum* amastigote activity (IC_50_ 3.2 and 3 μg/mL, respectively), solasodine only mild activity (IC_50_ 10.8 μg/mL), and hydroethanolic extract had no activity.	[46]
	Fruit	Alkaloid extract (44.4% of solasonine and 45.1% of solamargine)	C57BL/6 mice infected with *Leishmania mexicana* promastigotes treated topically with a formulation containing alkaloid extract (10 μmol/L each alkaloid) for 6 weeks	↓ Cutaneous lesion sizes and parasite counts recovered from lesions.	[28]
	Fruit	Alkaloid extract, solasonine, solamargine, and solasodine	In vitro antileishmanial activity against *Leishmania amazonensis* promastigotes	Solasodine was inactive. Alkaloid extract (IC_50_ 3.6 μg/mL), solamargine (IC_50_ 6.2 μmol/L), solasonine (IC_50_ 7.8 μmol/L), and equimolar mixture of solasonine and solamargine (IC_50_ 1.1 μmol/L) were highly active against *L. amazonensis* promastigotes after 72 h. Except for solasodine, the remaining samples exhibit no cytotoxicity against LLCMK_2_ cells.	[47]
	Fruit	Solasonine and solamargine isolated from lobeira	In vitro antileishmanial activity against *Leishmania mexicana* promastigotes and intracellular (mouse BMDM and (BMDDC) amastigotes forms	Solasonine and solamargine were more active against promastigote forms (IC_50_ 36.5 and 35.06 μmol/L, respectively) than reference drug sodium stibogluconate (IC_50_ 251.3 μmol/L). Solasonine and solamargine were more active against amastigotes forms inside BMDMs (IC_50_ 9.30 and 13.36 μmol/L, respectively) and BMDDCs (IC_50_ 5.93 and 6.03 μmol/L, respectively) than reference drug sodium stibogluconate (IC_50_ 14.32 and 47.91 μmol/L for BMDMs and BMDDCs, respectively).	[28]
	Fruit	Hydroethanolic extract (96% ethanol; 4.6% of solasonine and 4.4% of solamargine), hydroethanolic fraction (15.3% of solasonine and 35.7% of solamargine), solasonine (71.5%), and solamargine (63.1%)	In vitro antigiardial activity against *Giardia lamblia* trophozoites	Extract, fraction, and isolated compounds inhibited the growth of *G. lamblia* trophozoites (IC_50_ 13.23–120.30 μg/mL). Mixture of solasonine and solamargine (1:1) was the most potent giardicidal (IC_50_ 13.23 μg/mL) and showed the highest selectivity index.	[23]
	Fruit	Hydroethanolic extract (80% ethanol)	In vitro trypanocidal activity against *Trypanosoma cruzi* trypomastigotes	Extract induced the lysis of *T. cruzi* trypomastigotes (IC_50_ 57.1 μg/mL).	[48]
	Fruit	Hydroethanolic extract (96% ethanol) and solamargine	In vitro trypanocidal activity against *Trypanosoma cruzi* epimastigotes	Solamargine (IC_50_ 15.3 μg/mL) was more potent against *T. cruzi* epimastigotes than the crude extract (IC_50_ 194.7 μg/mL). Solamargine showed trypanocidal activity (IC_50_ 15.3 μg/mL) very close to the reference drug benznidazole (IC_50_ 9 μg/mL).	[49]
	Fruit	Alkaloid extract	Swiss mice infected with *Schistosoma mansoni* cercariae treated intragastrically with alkaloid extract (10, 20, and 40 mg/kg bw) for 5 days (between 37th and 41st day or between 45th and 49th day after infection)	Animals treated with the alkaloid extract (10 or 20 mg/kg bw) between the 37th and 41st day of infection showed an increased number of macrophages, elevated NO and IFN-γ concentrations, and reduced number of eggs and granulomas in the liver. Animals treated with the alkaloid extract between the 45th and 49th day of infection showed a reduced number of eggs (10 or 20 mg/kg bw) and granulomas (10–40 mg/kg bw) in the liver.	[47]
	Fruit	Alkaloid extract, solasonine, solamargine, and solasodine	In vitro schistosomicidal activity against *Schistosoma mansoni* eggs and adult worms	Alkaloid extract (10 and 15 μg/mL), solasonine (50 μmol/L), solamargine (10, 15, and 20 μmol/L), and equimolar mixture of solasonine and solamargine (10 and 15 μmol/L) reduced the development of eggs produced by the adult worms. Alkaloid extract (20, 32, and 50 μg/mL), solamargine (32 and 50 μmol/L), solasonine (50 μmol/L), and equimolar mixture of solasonine and solamargine (20, 32, and 50 μmol/L) caused the death of 100% of parasites, separation of 100% of couples, and extensive tegumental disruption, and reduced the motor activity within 24 h. Solasodine was inactive.	[50]
	Fruit	Solanine, solamargine, and solasodine isolated from lobeira fruit	In vitro antifungal activity against *Trichophyton rubrum* (ATTC MYA-3108)	Solamargine was the most potent alkaloid against *T. rubrum*, followed by solasodine and solanine (MIC of 3.12, 12.5, and >25 µg/mL, respectively). No effect on protoplasts regeneration and colony size of *T. rubrum*.	[51]
Anti-inflammatory	Ripe fruit	Ethanolic extract	Swiss mice treated intraperitoneally with 30, 100, and 300 mg/kg bw	↓ Carrageenan-induced acute inflammation in mouse footpads at 300 mg/kg bw. ↓ Carrageenan-induced tissue injury and migration of polymorphonuclear leukocytes to tissue at 300 mg/kg bw.	[18]
	Ripe fruit	Ethyl acetate and hydroethanolic fractions from ethanolic extract	Swiss mice treated intraperitoneally with 30, 100, and 300 mg/kg bw	Hydroethanolic fraction reduced carrageenan-induced acute inflammation in mouse footpads at 100 mg/kg bw (↓ paw edema). Ethyl acetate fraction had no anti-inflammatory effect at any of the doses evaluated.	[19]
	Ripe fruit	Dichloromethane fraction from ethanolic extract	Swiss mice treated intraperitoneally with 30, 100, and 300 mg/kg bw	↓ Carrageenan-induced acute inflammation in mouse footpads at all doses. ↓ Carrageenan-induced tissue injury and migration of polymorphonuclear leukocytes to tissue at 300 mg/kg bw.	[20]
	Fruit	Hydroethanolic extract (HE) (96% ethanol) and alkaloid fraction (AF)	Swiss mice treated intragastrically with 0.5, 1.0, and 2.0 g HE/kg bw or subcutaneously with 25, 50, and 100 mg AF/kg bw	HE and AF were able to inhibit the Croton oil-induced ear edema in a dose-dependent manner.	[4]
	Fruit	Alkaloid fraction	Swiss mice treated subcutaneously with 30, 100, and 300 mg/kg bw	↓ Carrageenan-induced total leukocyte migration to the peritoneum in a dose-dependent manner.	[4]
Antinociceptive	Ripe fruit	Ethanolic extract	Swiss mice treated intraperitoneally with 30, 100, and 300 mg/kg bw	↓ Acetic acid-induced nociception at all doses (↓ abdominal writhes). ↓ Formalin-induced nociception in both phases at 100 and 300 mg/kg bw (↓ paw licking time). ↑ Latency to response in the hot-plate test at 300 mg/kg bw.	[18]
	Ripe fruit	Ethanolic extract	Swiss mice treated intraperitoneally with 30, 100, and 300 mg/kg bw	↓ Acetic acid-induced nociception at all doses (↓ abdominal writhes). ↓ Formalin-induced nociception in the first phase at 100 and 300 mg/kg bw and the second phase at all doses (↓ paw licking time). ↑ Latency to response in the hot-plate test at all doses.	[20]
	Fruit	Hydroethanolic extract (96% ethanol)	Swiss mice treated intragastrically with 0.5, 1.0, and 2.0 g/kg bw	↓ Acetic acid-induced nociception in a dose-dependent manner (↓ abdominal writhes).	[4]
Antidiabetic	Fruit	Methanolic extract (ME) and its aqueous (WF), methanolic (MF), and acetonic (AF) fractions	Oral sucrose-loaded Wistar rats treated orally with 250 mg ME/kg bw, 100 mg WF/kg bw, 50 and 100 mg MF/kg bw, and 100 mg AF/kg bw	ME (250 mg/kg bw) and MF (100 mg/kg bw) reduced serum glucose levels in oral sucrose-loaded rats.	[25]
	Fruit	Solamargine and solasonine isolated from lobeira fruit	Oral sucrose-loaded Wistar rats treated orally with 25, 50, and 100 mg/kg bw and gastric emptying time in 1.5% CMC-Na-loaded mice treated orally with 25 and 50 mg/kg bw	Both alkaloids reduced serum glucose levels in oral sucrose-loaded rats in a dose-dependent manner. Solamargine suppressed the gastric emptying time in mice at a dose of 50 mg/kg, and solasonine also tended to suppress gastric emptying. Solamargine tended to have more potent effects than solasonine.	[25]
	Fruit	Calystegine-rich fraction	In vitro inhibitory activity against α-glucosidase	Calystegine-rich fraction (IC_50_ 49.06 μg/mL) showed higher inhibitory potential than the positive control acarbose (IC_50_ 59.07 μg/mL).	[32]

↑: increase, ↓: reduction, Bax: Bcl-2-associated protein X, Bcl-2: B-cell lymphoma 2, Bcl-xL: B-cell lymphoma extra-large, BHT: butylated hydroxytoluene, BMDDC: bone marrow-derived dendritic cell, BMDM: bone marrow-derived macrophage, MIC: minimum inhibitory concentration, bw: body weight, Cap: caspases, CMC-Na: carboxymethyl cellulose sodium salt, DMH: 1,2-dimethylhydrazine, DPPH: 2,2-diphenyl-1-picrylhydrazyl radical scavenging activity, FRAP: ferric-reducing antioxidant power, IC_50_: extract concentration that resulted in a 50% reduction in the enzymatic activity/cell proliferation or viability/radical concentration to the untreated control, IFN-γ: interferon gamma, MMC: mitomycin C, MMP: metalloproteinases, MMS: methyl methanesulfonate, NO: nitric oxide, ORAC: oxygen radical absorbance capacity, PARP: poly(ADP-ribose) polymerases, TE: Trolox equivalents, YVO_4_:Eu^3+^:CPTES:SM: nanoparticles of yttrium vanadate functionalized with 3-chloropropyltrimethoxysilane containing solamargine.

### 5.3. Antiparasitic Activity

Alkaloid-rich extracts and isolated alkaloids from different parts of the lobeira plant (leaves or fruits) have shown significant activity against human pathogens, including pathogenic fungi, protozoa, and worms (see Table 2). Cantelli et al. [51] evaluated the effect of three alkaloids isolated from the lobeira fruit (solanine, solamargine, and solasodine) against the dermatophyte fungus *Trichophyton rubrum*. The results demonstrated that solamargine was the most potent antifungal, followed by solasodine and solanine (MIC of 3.12, 12.5, and >25 µg/mL, respectively). However, none of the alkaloids were able to inhibit the protoplast regeneration and reduce the size of fungal colonies compared to the aculeacin control.

Hydroethanolic extract (96% ethanol; 4.6% solasonine and 4.4% solamargine), its hydroethanolic fraction (15.3% solasonine and 35.7% solamargine), and isolated alkaloids solasonine (71.5%) and solamargine (63.1%) from the lobeira fruit were tested against *Giardia lamblia* trophozoites. All treatments showed antigiardial activity (IC_50_ values of 13.23–120.30 μg/mL), but the hydroethanolic extract and the alkaloid solasonine were cytotoxic to macrophage (J774 cells) with IC_50_ values of 31.25 and 62.50 μg/mL, respectively. Interestingly, the mixture of the alkaloids solasonine and solamargine in equivalent proportions (1:1) was the most potent and selective giardicidal, with IC_50_ values of 13.23 and 250 μg/mL for *G. lamblia* trophozoites and macrophages, respectively [23].

Hydroethanolic extracts from lobeira fruit and the alkaloid solamargine were tested for their in vitro trypanocidal activities. Cunha et al. [48] found that the hydroethanolic extract of lobeira fruit (80% ethanol) induced the lysis of *Trypanosoma cruzi* trypomastigotes (IC_50_ value of 57.1 μg/mL) but was less potent than the positive control gentian violet (IC_50_ value of 31 μg/mL). In another study, Moreira et al. [49] evaluated the effect of the hydroethanolic extract of lobeira fruit (96% ethanol) and the alkaloid solamargine against *T. cruzi* epimastigotes. The results demonstrated that the extract had low trypanocidal activity (IC_50_ value of 194.7 μg/mL), while the alkaloid solamargine was a potent trypanocide (IC_50_ value of 15.3 μg/mL) whose effect was very close to the reference drug benznidazole (IC_50_ value of 9 μg/mL).

Alkaloid-rich extracts/fractions and isolated alkaloids from different parts of the lobeira plant (leaves and fruits) have been evaluated for their antileishmanial potential in both in vitro and in vivo models. Mans et al. [45] investigated the effect of leaf juice against promastigotes forms of *Leishmania guyanensis* (strain AMC2014), *L. major* (strain MHOM/IR/1972/NADIM5), and *L. donovani* (strain GEDII) and intracellular (THP-1 human acute monocytic leukemia cells) amastigotes form of *L. donovani* (strain BHU814). The authors verified that the extract was more active against promastigote forms, showing high activity against *L. donovani* GEDII promastigotes (IC_50_ value of 16 μg/mL) and low cytotoxicity on THP-1 cells (IC_50_ value > 500 μg/mL). However, the extract was only moderately active against *L. donovani* BHU814 amastigotes (IC_50_ value of 374 μg/mL). Clementino et al. [46] studied the antileishmanial activity of hydroethanolic extract from the lobeira fruit (96% ethanol; 4.6% solasonine and 4.4% solamargine), and alkaloids solasonine, solamargine, and solasodine against *L. infantum* promastigotes and amastigotes forms. Alkaloid solasodine was the most active compound against promastigote forms (IC_50_ value of 4.7 μg/mL), followed by solamargine, hydroethanolic extract, and solasonine (IC_50_ values of 8.1, 16.7, and 22.7 μg/mL, respectively). Alkaloids solasonine and solamargine showed high anti-*L. infantum* amastigote activity with IC_50_ values (3.2 and 3 μg/mL, respectively) very close to the positive control amphotericin B (2.3 μg/mL), whereas solasodine was only moderately active (IC_50_ value of 10.8 μg/mL), and hydroethanolic extract had no activity. Alkaloid solasonine presented the most promising results due to its high activity against amastigote forms and low cytotoxicity in murine macrophages, resulting in a higher selectivity index (3.7). In another study, Miranda et al. [47] evaluated the antileishmanial properties of the alkaloid extract from lobeira fruit and the alkaloids solasonine, solamargine, and solasodine against *L. amazonensis* promastigotes. Except for the alkaloid solasodine, the other treatments were active against *L. amazonensis* promastigotes after 72 h of incubation and were non-cytotoxic to LLCMK_2_ cells. Interestingly, the equimolar mixture of the alkaloids solasonine and solamargine was the most active antileishmanial agent (IC_50_ value of 1.1 μmol/L), slightly more potent than the positive control amphotericin B (IC_50_ value of 1.5 μmol/L). Furthermore, this alkaloid mixture was the most selective for *L. amazonensis* promastigotes due to its high antileishmanial activity and low cytotoxicity in LLCMK_2_ cells, resulting in a selectivity index of 9.1. Lezama-Dávila et al. [28] observed that the alkaloids solasonine (IC_50_ values of 35.06 and 6.03–13.36 μmol/L for promastigotes and amastigotes forms, respectively) and solamargine (IC_50_ values of 36.5 and 5.93–9.30 μmol/L for promastigotes and amastigotes forms, respectively) isolated from lobeira fruit were more active than the reference drug sodium stibogluconate (IC_50_ values of 251.3 and 14.32–47.91 μmol/L for promastigotes and amastigotes forms, respectively) against *L. mexicana* promastigotes and amastigotes forms. Both alkaloids showed high selectivity indices with values ranging from 9.3 to 20.2 and from 38.3 to 43.3 for *L. mexicana* amastigotes in bone marrow-derived macrophages (BMDM) and bone marrow-derived dendritic cells (BMDDC), respectively. Furthermore, the authors found that the topical application of a formulation containing alkaloid extract from lobeira fruit (10 μmol/L each alkaloid, i.e., solasonine and solamargine) for 6 weeks significantly delayed the growth of cutaneous lesions and reduced the number of parasites recovered from the lesions in mice infected with *L. mexicana* promastigotes.

The schistosomicidal activity of the alkaloid extract from lobeira fruit (about 90% of alkaloids) and its alkaloids solasonine, solamargine, and solasodine was evaluated against *Schistosoma mansoni* eggs and adult worms. Except for the alkaloid solasodine, the other treatments were effective against *S. mansoni*, causing parasite death, separation of couples, extensive tegumental disruption, and decreased motor activity of adult worms, in addition to reducing the development of eggs produced by adult worms [50]. In a subsequent study, Miranda et al. [47] evaluated the schistosomicidal effect of the alkaloid extract from lobeira fruit in mice infected with *S. mansoni* cercariae. Animals treated orally with alkaloid extract (10 or 20 mg/kg bw) between the 37th and 41st day of infection showed an increased number of macrophages, elevated NO and IFN-γ concentrations, and a reduced number of eggs and granulomas in the liver. On the other hand, animals that received the alkaloid extract between the 45th and 49th day of infection had a reduced number of eggs (10 or 20 mg/kg bw) and granulomas (10–40 mg/kg bw) in the liver. Thus, the alkaloid extract from the lobeira fruit may exert its schistosomicidal activity through its immunomodulatory effect.

So far, it has been demonstrated that lobeira alkaloids can be promising agents in the development of drugs for the treatment of various parasitic diseases, including mycoses, giardiasis, Chagas disease, leishmaniasis, and schistosomiasis.

### 5.4. Anti-Inflammatory Activity

Studies conducted in animal models have demonstrated the anti-inflammatory effect of alkaloid-rich extracts and/or fractions obtained from lobeira fruit (see Table 2). Vieira et al. [4] evaluated the anti-inflammatory effect of the hydroethanolic extract (96% ethanol) and the alkaloid fraction obtained from this extract in a Croton oil-induced ear edema model. In this study, mice were treated intragastrically with 0.5, 1.0, and 2.0 g hydroethanolic extract/kg bw or subcutaneously with 25, 50, and 100 mg alkaloid fraction/kg bw, and both treatments were able to inhibit the Croton oil-induced ear edema in a dose-dependent manner. Considering the positive effects of the alkaloid fraction at low concentrations, the authors also studied the anti-inflammatory effect of this fraction using the carrageenan-induced peritonitis model. Subcutaneous administration of the alkaloid fraction (30, 100, and 300 mg/kg bw) inhibited carrageenan-induced total leukocyte migration to the peritoneum in a dose-dependent manner. Morais et al. [18] investigated the anti-inflammatory effect of the ethanolic extract from ripe fruit (30, 100, and 300 mg/kg bw) in a carrageenan-induced paw edema model. The authors observed that intraperitoneal administration of 300 mg ethanolic extract/kg bw significantly reduced paw sole tissue injury and leukocyte infiltration into the dermis of mice. This extract was primarily composed of steroidal alkaloids (21 compounds) and phenolic compounds derived from caffeic and coumaric acids (8 compounds). In a subsequent study, this group assessed the anti-inflammatory activities of hexane, ethyl acetate, and hydroethanolic fractions obtained from the ethanolic extract of the ripe fruit in the same animal model. No anti-inflammatory activity was observed in mice treated intraperitoneally with the ethyl acetate fraction (30–300 mg/kg bw), while the hexane (100 and 300 mg/kg bw) and hydroethanolic (100 mg/kg bw) fractions significantly counteracted the carrageenan-induced paw edema. Instrumental analyses revealed that the hexane fraction was particularly composed of phytosterols stigmasterol and β-sitosterol, while the hydroethanolic fraction contained mainly steroidal alkaloids (10 compounds) and phenolic compounds derived from caffeic and coumaric acids (6 compounds) [19]. In a recent study, Morais et al. [20] evaluated the anti-inflammatory effect of the dichloromethane fraction obtained from the ethanolic extract using the same animal model. All administered doses (30–300 mg/kg bw) were able to inhibit paw edema formation by reducing carrageenan-induced leukocyte migration. Steroidal alkaloids (13 compounds) and phenolic compounds derived from caffeic and coumaric acids (4 compounds) were the main compounds found in this fraction. The alkaloids present in the lobeira fruit can prevent the triggering of inflammatory responses through their ability to scavenge free radicals and ROS/RNS, as well as inhibit leukocyte migration, consequently reducing the production and release of leukocyte-derived pro-inflammatory mediators (e.g., histamine, serotonin, bradykinin, prostaglandin, nitric oxide, and cytokines) [18,19,20].

### 5.5. Antinociceptive Activity

As shown in Table 2, some alkaloid-rich extracts obtained from lobeira fruit demonstrated analgesic effects in animal models. In addition to its anti-inflammatory activity, the hydroethanolic extract (96% ethanol) from the fruit exhibited analgesic effects when orally administered in mice, reducing acetic acid-induced abdominal writhes in a dose-dependent manner (0.5–2.0 g/kg bw). Furthermore, the lower dose of the extract tested (0.5 g/kg bw) showed antinociceptive activity comparable to the drug indomethacin (10 mg/kg bw), with inhibition percentages of abdominal constriction at 25.7% and 30.6%, respectively. However, this extract was inactive in the tail-flick analgesia test at both tested doses (1.0 and 2.0 g/kg bw, oral administration), suggesting that the extract does not contain opioid-like compounds with central analgesic properties [4]. Similar results were reported by Morais et al. [18] for the ethanolic extract of the ripe fruit tested in different analgesia models. In addition to attenuating the inflammatory process (see Section 5.4), oral administration of the extract reduced acetic acid-induced abdominal writhes (30, 100, and 300 mg/kg bw) and formalin-induced paw licking time (100 and 300 mg/kg bw), and increased latency to response in the hot-plate test in mice (300 mg/kg bw). Interestingly, all doses of the extract (30–300 mg/kg bw) were more effective than the standard drug indomethacin (10 mg/kg bw) in the acetic acid-induced abdominal writhing and formalin-induced nociception models. Furthermore, the extract at the highest tested dose (300 mg/kg bw) exhibited an antinociceptive effect similar to the standard drug morphine (7.5 mg/kg bw) in the hot-plate test. Twenty-one steroidal alkaloids and eight phenolic compounds derived from caffeic and coumaric acids have been identified in this extract [18]. In a subsequent study, Morais et al. [20] evaluated the antinociceptive effect of the dichloromethane fraction of this extract (30, 100, and 300 mg/kg bw) using the same animal models. The treatment with this fraction mitigated the inflammatory process (all doses) (see Section 5.4), reduced acetic acid-induced abdominal writhes (all doses) and formalin-induced paw licking time in the first (100 and 300 mg/kg bw) and second phases (all doses), and increased latency to response in the hot-plate test in mice (all doses). Remarkably, treatment with the dichloromethane fraction induced a greater effect than the standard drug indomethacin (10 mg/kg bw) in the acetic acid-induced abdominal writhing (300 mg/kg bw) and formalin-induced nociception (all doses) models. Additionally, all doses of this fraction displayed an antinociceptive effect similar to the standard drug morphine (7.5 mg/kg bw) in the hot-plate test. Instrumental analyses revealed the presence of 13 steroidal alkaloids and 4 phenolic compounds derived from caffeic and coumaric acids in this fraction [20]. Analgesic and anti-inflammatory effects of these extracts may be mediated, at least in part, by their alkaloids and phenolic compounds. These phytochemicals can act independently or synergistically by downregulating the expression of pro-inflammatory mediators, reducing oxidative stress, and modulating the peripheral and central nervous systems. Due to the ineffectiveness of pre-treatment with NaI (a non-selective opioid receptor antagonist) in reversing the antinociception caused by the ethanolic extract and its dichloromethane fraction in both phases of the formalin test, likely, the central antinociceptive mechanism of action of the compounds present in these extracts does not involve the participation of the opioidergic system [4,18,20].

### 5.6. Antidiabetic Activity

Alkaloid-rich extracts/fractions obtained from lobeira fruit and their alkaloids purified have shown promise in managing hyperglycemia (see Table 2). Souto et al. [32] investigated the in vitro inhibitory activity of α-glucosidase displayed by a calystegine-rich fraction obtained from lobeira fruit. This study demonstrated that the calystegine-rich fraction (IC_50_ value of 49.06 μg/mL) was a more efficient α-glucosidase inhibitor than the standard drug acarbose (IC_50_ value of 59.07 μg/mL). Four calystegines, namely, A_3_, B_1_, B_2_, and C_1_, were identified in the calystegine-rich fraction from lobeira fruit. Calystegine B_2_, recognized as a potent α-glucosidase inhibitor, was the major calystegine present in the lobeira fruit with a concentration of 48.34 mg/kg of fresh weight. A study conducted by Yoshikawa et al. [25] also demonstrated the potential antidiabetic effect of alkaloids isolated from lobeira fruit in vivo. Initially, the researchers evaluated the hypoglycemic effect of the methanolic extract of the fruit and its aqueous, methanolic, and acetonic fractions in oral sucrose-loaded rats, observing that only the methanolic extract (250 mg/kg bw) and its methanolic fraction (100 mg/kg bw) significantly reduced the serum glucose levels in rats. Five alkaloids were isolated and identified from the methanolic fraction: solamargine (0.42%), solasonine (0.67%), 12-hydroxysolasonine (0.0009%), robeneoside A (0.008%), and robeneoside B (0.0005%). Subsequently, the researchers assessed the hypoglycemic effect and mechanisms of action of the major alkaloids isolated from the methanolic fraction (i.e., solamargine and solasonine) using the same animal model. Both alkaloids significantly inhibited the increase in serum glucose levels in a dose-dependent manner (25–100 mg/kg bw), with the inhibitory effects of solamargine tending to be more potent than those of solasonine. Additionally, both alkaloids exhibited hypoglycemic effects similar, or even superior, to the drugs tolbutamide at 12.5–25 mg/kg bw (an insulin-secretion stimulant) and metformin at 125–500 mg/kg bw (an inhibitor of intestinal glucose absorption and enhancer of peripheral insulin sensitivity). Studying the hypoglycemic mechanism of action of solamargine and solasonine in a CMC-Na-loaded mice model, the researchers found that solamargine significantly suppressed the gastric emptying time in mice at a dose of 50 mg/kg bw, while solasonine tended to suppress gastric emptying time. Overall, it has been demonstrated that the hypoglycemic effect of lobeira fruit alkaloids may be mediated by the reduction in intestinal glucose absorption due to the inhibition of key digestive carbohydrase enzymes (e.g., α-glucosidase) [32] and the suppression of sucrose transfer from the stomach to the small intestine [25].

## 6. Conclusions

Lobeira fruit has attracted increasing interest from researchers around the world due to its use in folk medicine and documented biological properties. These beneficial effects have been associated with various bioactive phytochemicals present in the fruit, particularly alkaloids. The data collected here demonstrate that the alkaloids found in lobeira mainly belong to the classes of steroidal alkaloids and calystegines. Quantitatively, the steroidal glycoalkaloids solamargine and solasonine are the major alkaloids reported in lobeira fruit. However, the ripening stage of the fruits can affect the content of these alkaloids, with a reduction observed in their levels as the fruit matures. Recent studies compiled here found that alkaloid-rich extracts, obtained from different parts of the lobeira plant, can act as potent antioxidant, anti-inflammatory, anticancer, antigenotoxic, antinociceptive, antidiabetic, and antiparasitic agents. The presence of different alkaloids, mainly the steroidal glycoalkaloids solamargine and solasonine, can explain its biological effects obtained from in vitro assays and animal trials, as well as its effectiveness in folk medicine, demonstrating its promising potential for drug development to treat/manage various pathological conditions, including oxidative stress, inflammation, cancer, diabetes mellitus, pain, and illnesses associated to human parasites, including mycoses, giardiasis, Chagas disease, leishmaniasis, and schistosomiasis. Despite the strides made in identifying and quantifying alkaloid compounds from lobeira plant and determining their bioactivities, many scientific gaps remain to be filled. Unfortunately, the available literature is still limited, hindering the confirmation of health benefits in humans. In vivo studies are scarce, and no clinical trials have been undertaken to date. Consequently, it is imperative to conduct clinical and interventional studies involving humans to validate the biological effects observed in in vitro and in vivo studies and gain a clearer understanding of the actual advantages of lobeira alkaloids for human health and well-being. Additionally, meticulous toxicological investigations are essential for the isolated alkaloids and alkaloid-rich extracts/fractions from lobeira plant to establish the toxic dose and ensure the safety of subjects. The findings so far indicate that lobeira could serve as a valuable source of bioactive alkaloids with potential applications in foods, medicines, and cosmetics.

## Figures and Tables

**Table 1:** A summary of studies showing the alkaloid compounds found in lobeira fruit.

Plant Part	Extract Type	Major Findings	Ref.
Unripe and ripe fruits	Hydroethanolic extract (80% ethanol) and alkaloid extract (acid–base selective extraction)	Unripe fruits displayed higher concentrations of steroidal glycoalkaloids (1.04% solasonine and 0.69% solamargine) than ripe fruits (0.83% solasonine and 0.60% solamargine). Solasonine and solamargine contents in the alkaloid extract were superior to hydroethanolic extract (45.09% and 6.63% of solasonine and 44.37% and 4.35% of solamargine, respectively).	[8]
Unripe fruits and ripe fruits (peel, seeds, and pulp)	Hydroethanolic extract (70% ethanol)	Twenty-seven steroidal glycoalkaloids were identified in different fruit fractions and ripening stages (glycosylated forms derived from solasodine, solamargine, hydroxysolamargine isomers (Ring F), hydroxysolamargine isomers (Ring C), solasonine, unknown solasonine, hydroxysolasonine isomers (Ring F), hydroxysolasonine isomers (Ring C), dihydroxysolamargine isomers (Rings C, F), and dihydroxysolasonine isomers (Rings C, F). During the ripening, there was a reduction of almost 90% of solamargine in the pulp.	[17]
Ripe fruits	Ethanolic extract	Twenty-one steroidal alkaloid derivatives were putatively identified: 19 steroidal glycoalkaloids (robeneoside B or hydroxysolasonine isomers, solanandaine isomers, steroidal glycosylated alkaloid isomers, and khasianine or β_2_-solanine isomer) and 2 non-glycosylated steroidal alkaloids (peiminine and solasodine).	[18]
Ripe fruits	Ethanolic extract and its ethyl acetate and hydroethanolic fractions (70% ethanol)	Eleven steroidal alkaloid compounds were putatively identified in the ethyl acetate fraction: 9 steroidal glycoalkaloids (robeneoside B or hydroxysolasonine isomers, solanandaine isomers, steroidal glycosylated alkaloids, and khasianine or β_2_-solanine isomer) and 2 non-glycosylated steroidal alkaloids (peiminine and solasodine). Ten steroidal glycoalkaloid derivatives were putatively identified in the hydroethanolic fraction such as robeneoside B or hydroxysolasonine isomers, solanandaine isomers, steroidal glycosylated alkaloids, and khasianine or β_2_-solanine isomer.	[19]
Ripe fruits	Dichloromethane fraction from ethanolic extract	Thirteen steroidal alkaloid compounds were putatively identified: 10 steroidal glycoalkaloids (solasonine, solamargine, and unknown steroidal glycoalkaloids) and 3 non-glycosylated steroidal alkaloids (peiminine or imperialine, peimine or imperialine, and solasodine).	[20]
Ripe fruits	Hydroethanolic extract (80% ethanol)	Two steroidal glycoalkaloids were identified and quantified: solasonine (6.57%) and solamargine (4.60%).	[21,22]
Ripe fruits	Hydroethanolic extract (96% ethanol), its hydroethanolic fraction (40% ethanol), and isolated steroidal glycoalkaloids	In the hydroethanolic extract, steroidal glycoalkaloids solasonine and solamargine account for 4.6% and 4.4% of its composition, respectively. In the hydroethanolic fraction, these steroidal glycoalkaloids attained 15.3% and 35.7%, respectively. Isolated solasonine and solamargine had 71.5% and 63.1% of purity, respectively.	[23]
Ripe fruit	Hydroethanolic extract (70% ethanol) and alkaloid extract obtained by acid–base selective extraction	Five steroidal glycoalkaloids were identified (dihydroxysolamargine, 3 isomers of hydroxysolamargine, and solasonine).	[24]
Ripe fruits	Methanolic extract and its methanol-eluted fraction	Eleven steroidal glycoalkaloids were identified (lyconosides Ia, Ib, II, III, and IV, robeneosides A and B, solamargine, solasonine, 12-hydroxysolasonine, and lobofrutoside).	[6]
Ripe fruits	Methanolic extract and its methanol-eluted fraction	Five steroidal glycoalkaloids were identified (robeneosides A and B, solamargine, solasonine, and 12-hydroxysolasonine).	[25]
Ripe fruits	Alkaloid extract obtained by acid–base selective extraction	Two steroidal glycoalkaloids were identified and quantified: solasonine (42–45%) and solamargine (44–47%).	[26,27,28,29,30]
Ripe fruits	Alkaloid extract obtained by acid–base selective extraction	The steroidal glycoalkaloid solamargine was isolated and purified from the alkaloid extract.	[31]
Ripe fruits	Calystegines alkaloids rich fraction separated using an ion exchanger	Four polyhydroxy alkaloids were identified (Calystegines A_3_, B_1_, B_2_, and C_1_). The polyhydroxy alkaloid calystegine B_2_ was quantified (48.34 mg/kg fresh weight).	[32]

## Data Availability

The authors confirm that the data supporting the findings of this study are available within the article.
